# Temporal horns subependymomas: A report of two cases of an intraventricular neoplasm in an atypical location

**DOI:** 10.1259/bjrcr.20180068

**Published:** 2019-01-10

**Authors:** Monique Beraldo Ordones, Ana Cristhina Ribeiro Novaes, Carmen Lúcia Penteado Lancellotti, Lázaro Luís Faria do Amaral

**Affiliations:** 1 Department of Radiology, Hospital Beneficencia Portuguesa de Sao Paulo, Sao Paulo, Brazil; 2 Department of Neuropathology, Hospital Beneficência Portuguesa de São Paulo, São Paulo, SP, Brazil

## Abstract

Slow-growing intraventricular masses are sometimes imaging findings in asymptomatic patients. The neuroimaging characteristics frequently help making the correct diagnosis and the treatment decision. Subependymomas usually present as single lesions poorly vascularized, without invasion into adjacent brain parenchyma or cerebrospinal fluid dissemination. Ependymoma is considered the main differential diagnosis. We report two cases of this tumour who share the unusual location: The temporal horns. The lack of enhancement (or heterogeneous when present) and advanced neuroimaging techniques can be very useful in differentiating them from other lesions.

## CASE REPORT #1

### Clinical presentation

A 59-year-old female with history of incidental finding of brain tumor during examination for labyrinthitis 18 years ago. Patient returned after that time for further investigation. Review of systems: asymptomatic. Past medical history of arterial hypertension and diabetes. No epileptic seizures were associated. There were no neurological deficits or any other significant findings on physical examination or laboratory studies.

### Imaging findings

**Figure 1.**
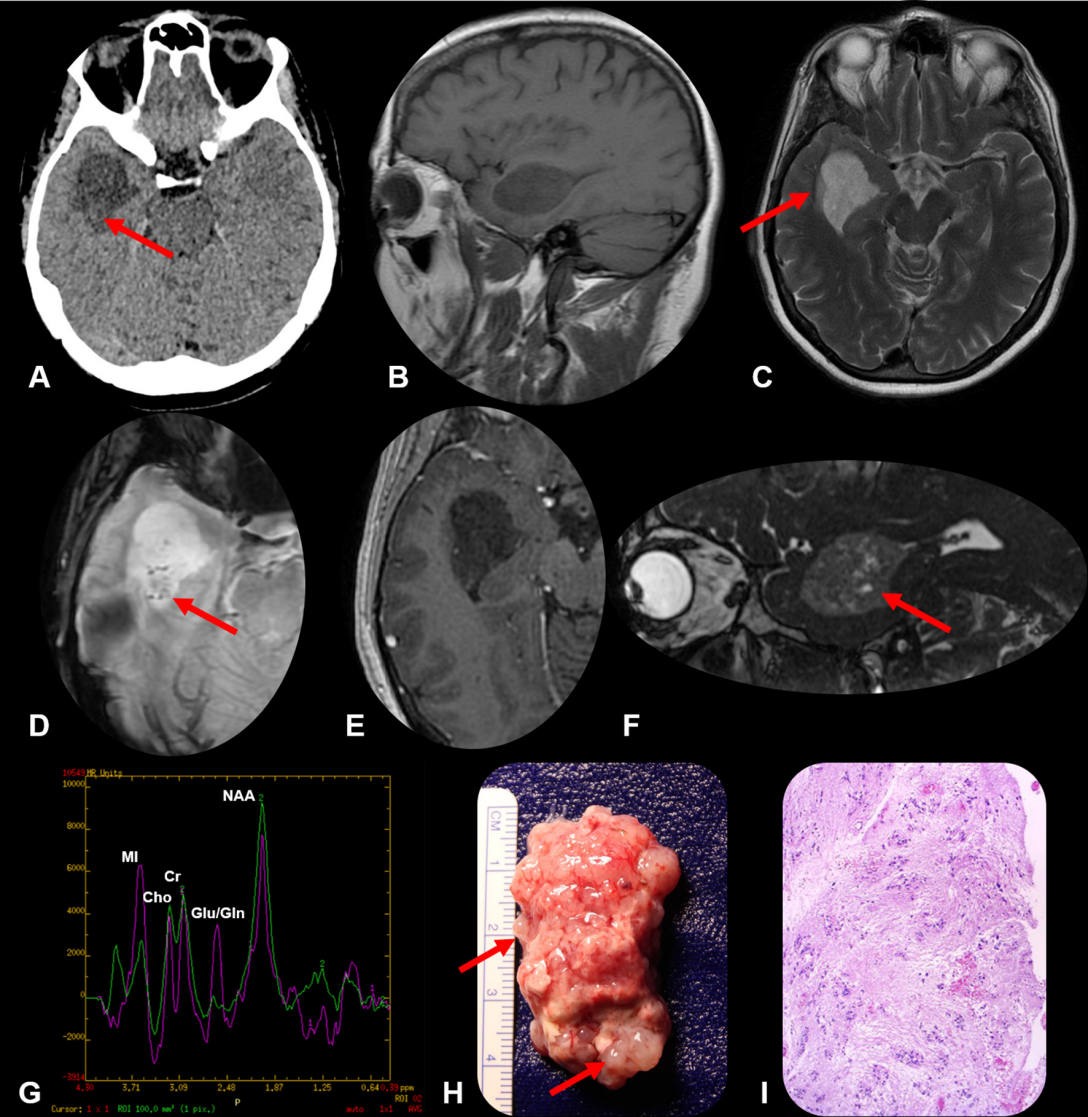
Non-contrast CT axial image shows a predominantly hypodense mass in the temporal horn of the right ventricle. Note there are tiny hyperdense foci (arrow), probably related to previous microhemorrhages (A) Sagittal *T*_1_ weighted MR image confirms a lobulated, well-defined intraventricular mass measuring 4.0 × 2.8 × 2.7 cm (AP × CC × LR), with no evidence of paraventricular extension. The mass is primarily hypointense to gray matter and causes expansion of the temporal horn of the right lateral ventricle. The third and fourth ventricles are of normal size. (B) Axial *T*_2_ weighted MR image shows the mass is heterogeneously hyperintense, and there is hyperintensity in the adjacent brain parenchyma of the temporal lobe, that might represent an edema (or infiltrative) component (arrow) (C) Axial SWI shows small hypointense foci inside the lesion, corresponding to microhemorrhages (D) There was no or minimal enhancement post-gadolinium (E) Sagittal 3D-CISS (FIESTA) images show a heterogeneous high-signal-intensity solid lesion, with cystic components within the lesion (arrow) (F) Short TE 3D Multi voxel spectroscopy demonstrated the NAA level partially decreased, due to neuronal viability loss, normal peak of Choline, and an increased peak of myoinositol, meaning astrogliosis, compared to the normal left temporal lobe (G) The macroscopic specimen obtained at surgical resection correlates with the imaging findings: A lobulated mass, with heterogeneous consistency, containing small cysts (arrows) (H). Histological slices with H&E staining reveal a paucicellular neuroepithelial neoplasm, characterized by small clusters of glial cells, with isomorphic vesicular nuclei (I) NAA, N-acetylaspartate; WI, susceptibility weighted-image; TE, echo time.

Head CT revealed an intraventricular expansile mass, which caused a right ventricular temporal horn expansion (Figure 1A). Brain MRI showed the lesion was lobulated, well-defined, heterogeneous and with cystic components within the lesion (Figure 1F). Hypointensities were also seen in the susceptibility weighted-image (SWI)-weighted MR sequence, related to hemosiderin deposits by previous microhemorrhages (Figure 1D).

A subtle hyperintensity in the adjacent brain parenchyma was noted in the temporal lobe, that might represent an edematous (or infiltrative) component (Figure 1C).

The Protons Spectroscopy with short TE demonstrated a decreased N-acetylaspartate (NAA) peak, suggesting neuronal viability loss, and an increased peak of myoinositol (MI), meaning astrogliosis. The Choline (Cho) peak remained normal, since there wasn’t an increase in the capillary density (Figure 1G).

Thus, a diagnosis of a primary glial low-grade central nervous system neoplasm (subependymoma) was considered as the most likely possibility.

## Treatment and outcome

The patient insisted on a surgical approach and underwent a right frontotemporosphenoidal craniotomy. The lesion was poorly vascularized, and a complete macroscopic tumor removal was achieved.

Post-operative tumor histopathology revealed a paucicellular neuroepithelial neoplasm, with small clusters of glial cells containing isomorphic vesicular nuclei, although varied sizes of nuclei were also present. The tumor cells were loosely arranged in a fibrillary matrix, with sparse microcysts and ependymal lining, typical features of subependymoma (Figure 1H and I).

Histological features, such as increased cellularity, nuclear atypia, necrosis and microvascular proliferation were analyzed, as well as immunohistochemistry was performed using antibodies for GFAP, Synaptophysin, S100 and EMA. There was a positive expression for S100 and GFAP. Ki-67 immunohistochemistry was also performed, and the proliferation index was low (Ki67 <1%). The final report considered it a Subependymoma (WHO grade I).

The postoperative brain MRI showed complete resection of the intraventricular lesion and there was no evidence of disease at the last follow-up.

## CASE REPORT #2

### Clinical presentation

A 22-year-old female with history of headache with sensation of pressure on the sides and across the forehead, and blurred vision. The headache would persist over a period of several hours. Her past medical history was otherwise unremarkable. No epileptic seizures were associated. She had no sick contacts or recent travel history. Her general and neurologic examinations did not show any neurological deficits. A brain MRI was performed for investigation and revealed the intraventricular lesion in the left temporal horn.

### Imaging findings

**Figure 2.**
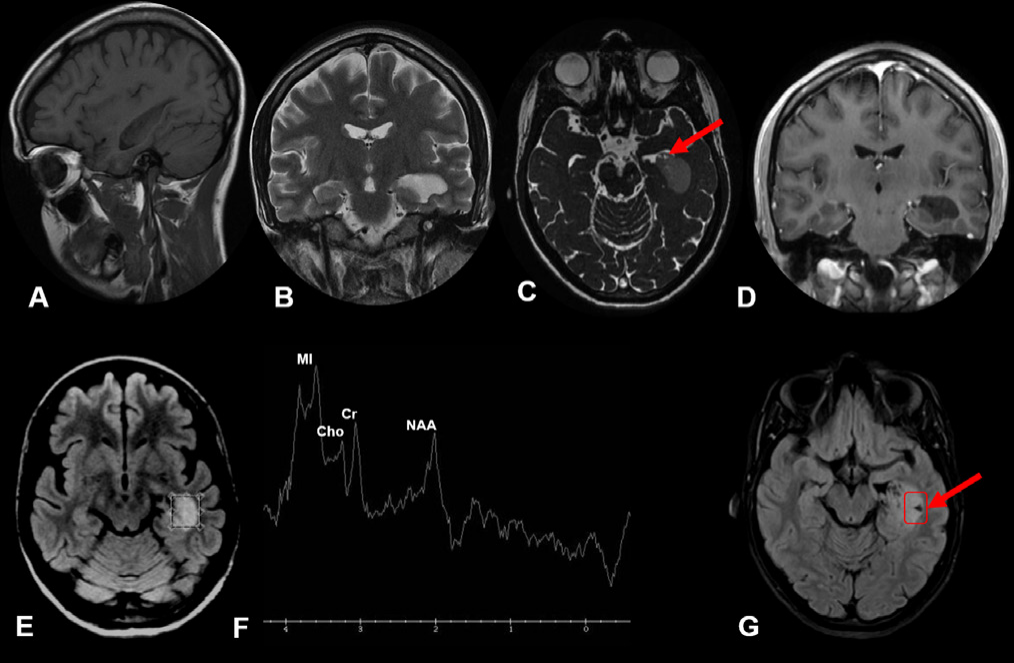
Sagittal *T*_1_ weighted MR image shows an isointense lobulated mass, in the left temporal horn of the lateral ventricle (A). Coronal T2 weighted MR image shows an iso/hyperintense solid lesion (B). Axial 3D-CISS (FIESTA) image shows heterogeneous hyperintensity within the tumors and confirm its intraventricular location (C). Post-contrast Coronal 3D-MPRAGE shows no enhancement (D). Short TE 3D Uni-voxel spectroscopy demonstrated a NAA peak partially decreased, due to neuronal viability loss and an increased peak of myoinositol, meaning astrogliosis (E, F). Axial FLAIR image shows the tumor after biopsy (arrow) (G). FLAIR, fluid attenuation inversion recovery; NAA, N-acetylaspartate; WI, susceptibility weighted-image; TE, echo time.

Brain MRI revealed a lobulated solid lesion in the left ventricular temporal horn, isointense in *T*_1 _weighted imaging (Figure 2A) and hyperintense in *T*_2 _weighted imaging (Figure 2B). No enhancement was seen in the post-gadolinium images (Figure 2D). The 3D-CISS (FIESTA) sequence images showed heterogeneous hyperintensities within the lesion (cystic components) and confirmed its intraventricular location (Figure 2C).

Short echo time (TE) 3D Multi voxel spectroscopy demonstrated a NAA peak partially decreased, due to neuronal viability loss and an increased peak of myoinositol, meaning astrogliosis (Figure 2F).

### Treatment and outcome

A brain tumor surgical biopsy was performed in the left temporal region, without any procedural complications (Figure 2G).

Histopathology revealed a hypocellular neuroepithelial neoplasm, with clustering cells containing rounded vesicular nuclei and delicate chromatin. The tumor cells were dispersed in a loose fibrillary matrix, often arranged in perivascular pseudorosettes. There were no histological signs of malignancy. The features were typical of a subependymoma.

### Differential diagnosis

The differential diagnosis for intraventricular lesions includes subependymoma, ependymoma, neurocytoma, and giant cell subependymal astrocytoma. When there is microcystic degeneration, the list should contain pilocytic astrocytomas, chordoid gliomas, myxoide meningioma and oligodendrogliomas. Immunohistochemistry may help, when it shows strong immunopositivity for GFAP and S-100 antigens.

Here we discuss the three main differential diagnoses, starting with ependymoma. The imaging appearance may be similar, as both lesions are usually intraventricular, and calcification, hemorrhage, and cystic components can be seen, although other findings help in the differentiation process. Subependymomas frequently present in the fifth or sixth decade of life, and although ependymomas may occur at any age, the posterior fossa tumors tend to present more commonly in the pediatric age group. Intense enhancement is observed in ependymomas, as well as extraventricular extension to the brain parenchyma and the cerebrospinal fluid (CSF). Subependymomas may show no or subtle enhancement and there is usually no invasion into the adjacent brain parenchyma (only peritumoral edema) nor CSF dissemination. Curiously, when there is overlap of these imaging features, the lesion tends to have an ependymoma-like clinical course, therefore it is graded on the basis of the ependymoma component. Those cases are referred to as mixed ependymoma–subependymoma, and represent the main problem in the histological diagnosis.

Another differential diagnosis is neurocytoma, which is typically seen in young adults (nearly 70% diagnosed between 20 and 40 years of age), emerging from the septum pellucidum or the ventricular wall, and lateral ventricles involvement is seen in half of the cases, near the foramen of Monro, while 15% are located in both the lateral and third ventricles. They may also contain calcifications and cysts within the lesion, with mild enhancement. Spectroscopy may be helpful, as subependymomas show a large MI peak (produced only by astrocyte cells), not seen in neurocytomas.^1^

Finally, giant cell astrocytomas are generally present in the context of a patient with tuberous sclerosis, at a younger age (most cases occurring in the first and second decades), with a mean age of 11 years. Imaging findings include calcifications and the typical location, virtually always located near the foramen of Monro.

## Discussion

Subependymomas are slow-growing benign neuroepithelial neoplasms (WHO Grade I). They are usually incidental findings in middle-aged patients, during neuroimaging investigation for other reasons. No race or gender predilection is known.^2–4^ The most common locations are the fourth and lateral ventricles.^5–7^

Patients with subependymoma are frequently asymptomatic. When symptomatic, they usually present with hydrocephalus and the lesion is most often greater than 2.0 cm.^7^

Most subependymomas present as a well-circumscribed macrolobulated intraventricular mass, that is hypo/isodense to brain parenchyma at CT (Figure 1A), with cystic components (Figures 1F and 2C). Usually there is no periventricular extension and minimal or no contrast enhancement at all^5,6,8,9^ (Figures 1E and 2D).

MR imaging shows similar findings: the lesion is typically hypo/isointense on *T*_1_ weighted imaging (Figure 1B e 2A) and hyperintense on *T*_2_ weighted imaging (Figure 1C e 2B). The cystic components are better seen and confirmed in the 3D-CISS (FIESTA) sequence (Figures 1F and 2C). In the minority of cases where there is contrast medium enhancement, it is typically heterogeneous.^4,5,8,9^

Although rare, peritumoral edema, high vascularity, intratumoral hemorrhage (Figure 1A and D), calcifications and mass effects may occur.

In the protons spectroscopy study, the subependymoma have large MI peak (Figures 1G and 2F). MI is an important osmolyte in neuroglial cells and accumulates intracellularly after cell shrinkage to maintain cell volume homeostasis and can be seen in low grade glial lesions.

The histological features of subependymoma are low cellular density, and small clusters of glial cells, dispersed in a loose fibrillary matrix, near the ependymal lining.^3,7,8^ There are also small isomorphic vesicular nuclei and microcystic degeneration (Figure 1H). Mitosis is usually not observed, as there is no evidence of atypia or necrosis. Although rare, sometimes ependymal true rosettes can be seen.^10^

## Learning points

1. The reported cases illustrate intraventricular masses at an unusual location of the lateral ventricles: The temporal horns.
2. Subependymomas are usually single lesions in the fourth and lateral ventricles in patients frequently asymptomatic. When the lesion is greater than 2.0 cm, it can cause obstruction of the CSF circulation, causing hydrocephalus.
3. CT shows a well-circumscribed macrolobulated intraventricular mass, that is heterogenous, hypo/isodense to brain parenchyma. Usually there is no periventricular extension and no contrast enhancement.
4. MRI of both cases show hypo/isointense lesion on *T*_1 _weighted imaging and hyperintense on *T*_2 _weighted imaging. Cystic degeneration may occur. If there is contrast enhancement, it is typically tenuous and heterogeneous.
5. Histological features include low cellular density and small clusters of glial cells, dispersed in a loose fibrillary matrix, near the ependymal lining, considered a benign neuroepithelial neoplasm (WHO Grade I). Recurrence is very rare.

## References

[1] Ueda F , Aburano H , Ryu Y , Yoshie Y , Nakada M , Hayashi Y , et al . MR Spectroscopy to Distinguish between Supratentorial Intraventricular Subependymoma and Central Neurocytoma . Magn Reson Med Sci 2017 ; 16 : 223 – 30 . doi: 10.2463/mrms.mp.2015-0013 27941295 PMC5600029

[2] Rath TJ , Sundgren PC , Brahma B , Lieberman AP , Chandler WF , Gebarski SS . Massive symptomatic subependymoma of the lateral ventricles: case report and review of the literature . Neuroradiology 2005 ; 47 : 183 – 8 . doi: 10.1007/s00234-005-1342-3 15702322

[3] Maiuri F , Gangemi M , Iaconetta G , Signorelli F , Del Basso De Caro M . Symptomatic subependymomas of the lateral ventricles. Report of eight cases . Clin Neurol Neurosurg 1997 ; 99 : 17 – 22 . doi: 10.1016/S0303-8467(96)00554-9 9107462

[4] Akamatsu Y , Utsunomiya A , Suzuki S , Endo T , Suzuki I , Nishimura S , et al . Subependymoma in the lateral ventricle manifesting as intraventricular hemorrhage . Neurol Med Chir 2010 ; 50 : 1020 – 3 . doi: 10.2176/nmc.50.1020 21123990

[5] Shogan P , Banks KP , Brown S . *AJR* teaching file: Intraventricular mass . AJR Am J Roentgenol 2007 ; 189 ( 6_supplement ): S55 – S57 . doi: 10.2214/AJR.07.7027 18029902

[6] Osborn AG “Introdução a neoplasias, cistos e lesões pseudotumorais” In: Artmed Osborn's Brain: Imaging, Pathology, and Anatomy. 1st ed; 2014.

[7] Kurukumbi M , Muley A , Ramidi G , Wynn Z , Trouth AJ , Jayam Trouth A . A rare case of subependymoma with an atypical presentation: a case report . Case Rep Neurol 2011 ; 3 : 227 – 32 . doi: 10.1159/000333061 22121350 PMC3223030

[8] Koeller KK , Sandberg GD , Armed Forces Institute of Pathology . From the archives of the AFIP. Cerebral intraventricular neoplasms: radiologic-pathologic correlation . Radiographics 2002 ; 22 : 1473 – 505 . doi: 10.1148/rg.226025118 12432118

[9] Castro FDde , Reis F , Guerra JGG . Lesões expansivas intraventriculares ressonância magnética: ensaio iconográfico - parte 1 . Radiologia Brasileira 2014 ; 47 : 176 – 81 . doi: 10.1590/0100-3984.2013.1696 25741075 PMC4337139

[10] You H , Kim YI , Im SY , Suh-Kim H , Paek SH , Park SH , et al . Immunohistochemical study of central neurocytoma, subependymoma, and subependymal giant cell astrocytoma . J Neurooncol 2005 ; 74 : 1 – 8 . doi: 10.1007/s11060-004-2354-2 16078101

